# Comparative Transcriptome Analysis Revealing the Potential Salt Tolerance Mechanism of Exogenous Abscisic Acid Application in *Melilotus albus*

**DOI:** 10.3390/ijms252413261

**Published:** 2024-12-10

**Authors:** Lijun Chen, Fan Wu, Zhen Duan, Shengsheng Wang, Yuncan Qu, Bao Ao, Xiaojuan Sun, Jiyu Zhang

**Affiliations:** State Key Laboratory of Herbage Improvement and Grassland Agro-Ecosystems, Key Laboratory of Grassland Livestock Industry Innovation, Ministry of Agriculture and Rural Affairs, College of Pastoral Agriculture Science and Technology, Lanzhou University, Lanzhou 730020, China; chenlj16@lzu.edu.cn (L.C.); wufan@lzu.edu.cn (F.W.); duanzh12@lzu.edu.cn (Z.D.); wangshsh2015@lzu.edu.cn (S.W.); quyc2023@lzu.edu.cn (Y.Q.); aob20@lzu.edu.cn (B.A.); sunxj2024@lzu.edu.cn (X.S.)

**Keywords:** *Melilotus albus*, exogenous ABA, transcriptomics, WGCNA, *Ma4CL1*, salt tolerance

## Abstract

*Melilotus albus*, which contains abundant pharmacologically active coumarins, is usually used as a rotation crop and green manure worldwide. Abscisic acid (ABA) is a crucial plant hormone that plays an important role in plant stress responses. There is a paucity of information about the ABA signaling pathway and its regulatory network in *M. albus*. Here, we performed a comparative physiological and transcriptomic analysis to assess the response of *M. albus* to exogenous ABA. Physiological analysis revealed that proline (Pro), soluble protein and H_2_O_2_ content after ABA treatment 3 h significantly increased by 14.0%, 12.0% and 32.4% compared with 0 h in *M. albus*. A total of 19,855 differentially expressed genes (DEGs) were identified under ABA treatment, including 13,392 in shoots and 15,471 in roots. We obtained two modules that were significantly correlated with the ABA treatment (the darkorange module was positively correlated at 24 h in the shoot, brown2 module positively correlated at 3 h in the root) by weighted correlation network analysis (WGCNA). KEGG enrichment analysis showed that genes within two modules were primarily enriched in protein synthesis and metabolism, secondary metabolites, purine and pyrimidine metabolism, and phenylalanine, tyrosine and tryptophan biosynthesis. GO enrichment analysis indicated that genes within two modules were primarily enriched in energy substance metabolism. These pathways were mainly associated with abiothic stress, which indicated that exogenous application of ABA activated the stress resistance system of *M. albus*. The hub gene *4CL1* (4-Coumarate: CoA ligase 1) was translated and expressed in yeast, resulting in enhanced salt and ABA tolerance in the transgenic yeast. Overexpression of *Ma4CL1* in *M. albus* improved the salt resistance of the transgenic plants. Profiling ABA-responsive genes offers valuable insights into the molecular functions of regulatory genes and will facilitate future molecular breeding efforts in *M. albus*.

## 1. Introduction

*Melilotus*, commonly known as wild alfalfa or sweet clover, encompasses approximately 19 species that are either annual or biennial. They are widely dispersed across North Africa, subtropical Asia, the Mediterranean, and temperate Europe. In the first half of the 20th century, sweet clover was regarded as the king of green manure and grazing legume in the southern and midwestern regions of the United States [1]. Moreover, *Melilotus* is renowned as a medicinal plant due to its high concentrations of the secondary plant compound coumarin [2]. Species belonging to the genus *Melilotus* have garnered widespread attention for their remarkable adaptability to extreme environments, such as saline, cold and drought conditions [3,4]. Interestingly, coumarin content in the plant is associated with adversity. It has been reported that the coumarin content of *M. albus* increased with the intensification of drought and low temperature stress [5]. *M. albus* is a common species in the genus *Melilotus*, and its whole genome sequence and transcriptomic data under salt and drought stress are available [6,7].

Abscisic acid (ABA) is a pivotal phytohormone which plays a vital role in regulating plant growth and development, and it is recognized as a stress hormone that modulates numerous essential biological processes [8,9]. ABA plays a crucial role in various plants by bolstering their resilience to various stressors, including cold, heat, drought, and salinity [10,11]. There were many transcriptome datasets showed that ABA was involved in regulating and enhancing the levels of secondary metabolites [12,13]. For example, studies have demonstrated that exogenous ABA treatment boosts the genes expression responsible for flavonoid and carotenoid biosynthesis in tomato fruits [14]. Furthermore, it has been found that applications of ABA could stimulate the synthesis of monoterpenes and anthocyanins in grapes [15]. Plants have developed distinct strategies to adapt and respond to detrimental environmental signals by altering gene expression and metabolic activity [16]. For example, the accumulation of proline (Pro) may prevent water loss, as both antioxidant activity and Pro content increased in apples under osmotic stress [17]. A previous study suggested that ABA partially regulated the accumulation of Pro [18]. Pre-treatment with ABA assists plants in conserving the energy necessary for their survival under adverse environmental conditions [19]. Phytohormones production could be altered by abiotic stresses, and consequently, these hormones can mediate stress responses via the actions of hormones and stress responses transcription factors [20].

An exogenous application of ABA had a significant positive impact on the activities of 4-Coumarate, coenzyme A ligase (4CL) enzymes [21]. 4CL served as a crucial rate-limiting enzyme within the phenylalanine metabolic pathway, played a pivotal role in the biosynthesis of coumarins, flavonoids, phenylpropanoids and so on in plants [22,23,24]. Members in 4CL gene family contains both the AMP-binding enzyme domain (AMP-binding; PF00501) and the AMP-binding enzyme C-terminal domain (AMP-binding_C; PF13193) [25]. In plants, the *4CL* genes participated in responding to a number of abiotic stresses [26]. The overexpression of the *Fraxinus mandshurica* 4CL gene in tobacco led to a significant increase in content of lignin, and enhanced the drought tolerance of the transgenic tobacco plants [27]. The promoter region of *4CL* contained multiple cis-acting elements that were associated with phytohormones and stress responses, and presented a positive regulation mode after salt stress in walnuts [28]. Ethylene response factors (ERFs) served as a pivotal regulatory center, integrating signals from redox abscisic acid and ethylene processes in response to various abiotic stresses in plants [29]. Exogenous application of ABA has resulted in the induction of expression in several ERF genes including *AtERF1*, *GhERF2*, *AtERF4*, *JcERF1* [29,30,31]. Overexpression of *ERF1* in *Arabidopsis* enhanced resistance to salt stresses, accompanied by elevated levels of ABA and Pro [32]. Overexpression lines and knockout insertion mutants of *Arabidopsis* demonstrated the role of *ERF109* in improving salt tolerance [33]. *ERF34* enhanced salt resistance at various stages of plant growth, and played an important role in the integration of salt stress responses with leaf senescence programs [34].

Transcriptome sequencing enables the effective determination of gene expression profiles on genome wide scale, making it a widely utilized method for identifying crucial genes linked to significant traits. In our previous studies, based on lncRNAs and miRNA transcripts analysis, WD40 and miR7696a-3p were identified, which may have been due to combined regulated *M. albus* salt tolerance [7]. The uridine diphosphate (UDP) glycosyltransferase (UGT) gene family was analyzed using genome and drought stress transcriptome data, and the overexpression of *MaUGT79* resulted in enhanced drought resistance of *M. albus* [6,35,36]. However, there have been limited reports on RNA-seq analyses that have investigated the genome-wide responses of genes to ABA in *M. albus*. Considering the pivotal role of ABA in plant development and stress responses, it is highly necessary to understand the potential mechanism of the ABA regulatory network at the transcriptional level in *M. albus*. In the present study, we investigated ABA responses in *M. albus*, reported the findings of a comparative transcriptome analysis of *M. albus* shoots and roots treated with exogenous ABA using RNA-seq technology, and functionally characterized the *Ma4CL* gene that is potentially responsive to stress tolerance in vitro and in vivo. This study offers implementation strategies for investigating potential key genes involved in the ABA signaling pathway and stress responses in *M. albus*. The resources established here provide a genetic resource for *M. albus* and other crop improvement.

## 2. Results

### 2.1. Physiological Responses of M. albus Under ABA Treatment

**Figure 1:**
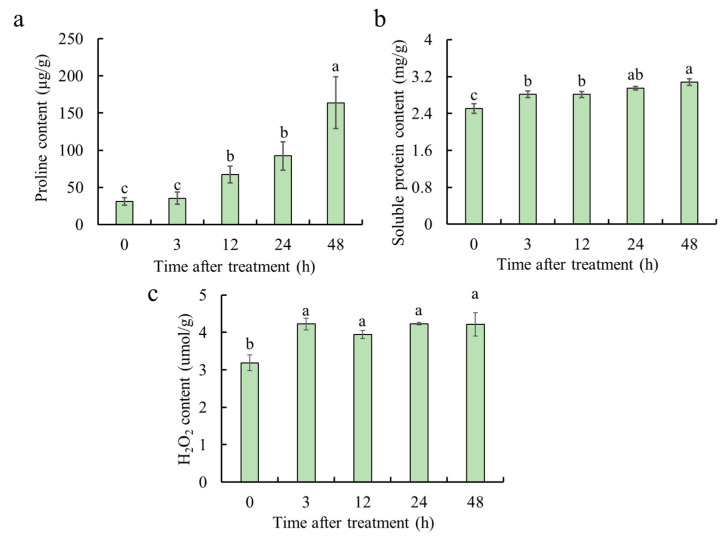
Physiological analysis was conducted on *M. ablus* in response to ABA treatment. The contents of proline (**a**), soluble protein (**b**) and H_2_O_2_ (**c**) in the *M. albus* shoot were measured at 0 h, 3 h, 12 h, 24 h and 48 h after ABA treatment. The six-week-old *M. albus* plants were applied to 100 μM ABA. Different lowercase letters represented significant differences at *p* < 0.05 level.

To investigate the physiological response of *M. albus* to ABA treatment and to further determine the sampling time for transcriptome analysis, three physiological indicators were measured in the shoots at 0 h, 3 h, 12 h, 24 h and 48 h time-points after ABA treatment, respectively. Compared with that at 0 h, the content of proline significantly increased by 14.0% and 199.0% after 3 h and 24 h of ABA treatment, respectively (Figure 1a). Under ABA treatment, the soluble protein content stably increased as time passed; in comparison with 0 h, the soluble protein content at 3 h and 24 h was increased by 12.0% and 17.6%, respectively (Figure 1b). H_2_O_2_ content increased significantly 3 h after ABA treatment and subsequently maintained at a stable level, whereas the H_2_O_2_ content at 3 h increased by 32.4% compared with that at 0 h (Figure 1c). Considering the changes in the physiological indexes under ABA treatment, shoot and root samples of 0 h, 3 h and 24 h were subjected to transcriptome sequencing analysis.

### 2.2. Comparative Transcriptome Analysis of M. albus to ABA Treatment

To investigate the gene expression patterns of genes in *M. albus* seedlings under ABA treatments, high-throughput sequencing was performed for a total of 18 samples. An amount of 207 Gb of clean data were obtained after removing low-quality and contaminated reads which were mapped to *M. albus* genome (Supplementary Table S1). The results of the data quality assessment revealed that the Q30 and the reads mapping rate were mostly greater than 92 and 85%, respectively (Supplementary Table S1).

**Figure 2:**
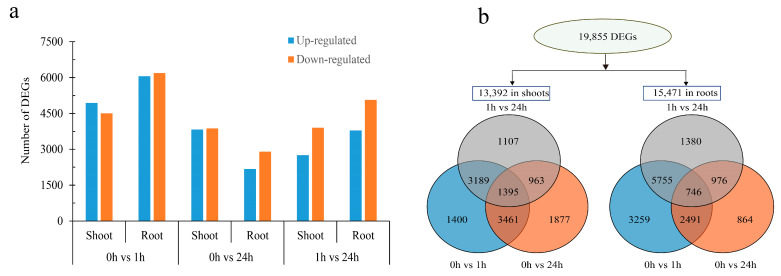
Summary of DEGs in *M. ablus* in response to ABA treatment. (**a**) DEGs were identified by comparing the gene expression between the two groups in shoots and roots under ABA treatment. (**b**) The Venn diagram illustrates the overlapping DEGs under different ABA treatment times in shoots and roots.

Overall, the gene FPKM values of each sample conform to a normal distribution (Supplementary Figure S1). The FPKM value of genes in the ABA-treated groups were compared with that of control groups. Volcano plots were utilized to depict the overall alterations in expression level of genes between two groups (Supplementary Figure S2). The results suggested that number of genes exhibiting significant changes were expressed differently in different combined groups. We identified 9445 DEGs (the proportion of up-regulated was 52.3%, and down-regulated was 47.7%) in the shoot and 12,251 DEGs (up-regulated 49.4%, down-regulated 40.6%) in the root at 0 h vs. 3 h (Figure 2a). The number for down-regulated DEGs was greater than that of up-regulated in both shoots and roots at 0 h vs. 24 h and 3 h vs. 24 h (Figure 2a). In total, 19,855 genes were significantly differentially expressed under ABA treatment (Figure 2b). Among these genes, the number of DEGs in the roots was 15.5% greater than that in the shoots (13,392 in shoots and 15,471 in roots). In the shoots, there were 1400 DEGs at 0 h vs. 3 h, 1877 DEGs at 0 h vs. 24 h, and 1107 DEGs at 3 h vs. 24 h. Compared with 0 h vs. 3 h, the DEGs at 0 h vs. 24 h increased by 34.1%, and DEGs at 3 h vs. 24 h decreased by 20.9%. In addition, in roots, DEGs at 0 h vs. 24 h in the comparison group and 3 h vs. 24 h in the comparison group decreased by 73.5% and 57.7% compared with 0 h vs. 3 h, respectively. Furthermore, 1395 and 746 DEGs overlapped in three comparison groups in the shoots and roots.

### 2.3. Weighted Correlation Network Analysis (WGCNA)

**Figure 3:**
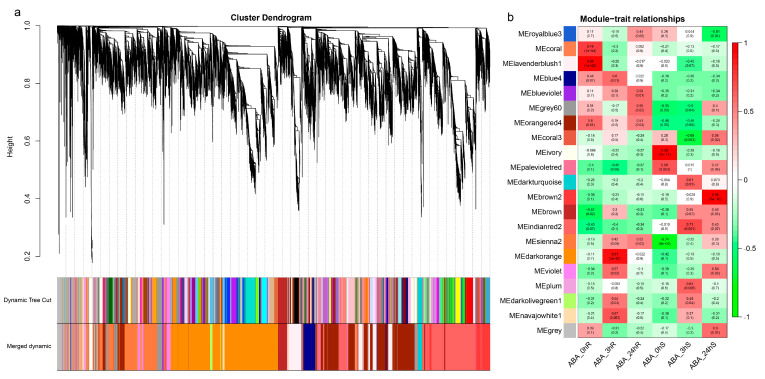
A dendrogram from the network analysis showed the modules identified through weighted correlation network analysis (WGCNA). (**a**) A dendrogram plot featuring color annotations. (**b**) Weight correlations between the module and ABA treatment, along with their corresponding *p*-values. The column on the left displayed 21 modules and their corresponding colors. The color scale on the right showed the correlation between modules and traits from −1 (green) to 1 (red).

WGCNA was performed to identify the genes that were responsive to ABA treatment in *M. albus* (Figure 3a). The weighted gene co-expression network was constructed, and the network consisted of 21 modules which were named based on their corresponding color (Figure 3b). The number of genes in these modules varied from 47 (royalblue3 module) to 5831 (darkorange module). The correlation coefficients between the modules and the treatment time varied from −0.74 to 0.98 (Figure 3b).

**Figure 4:**
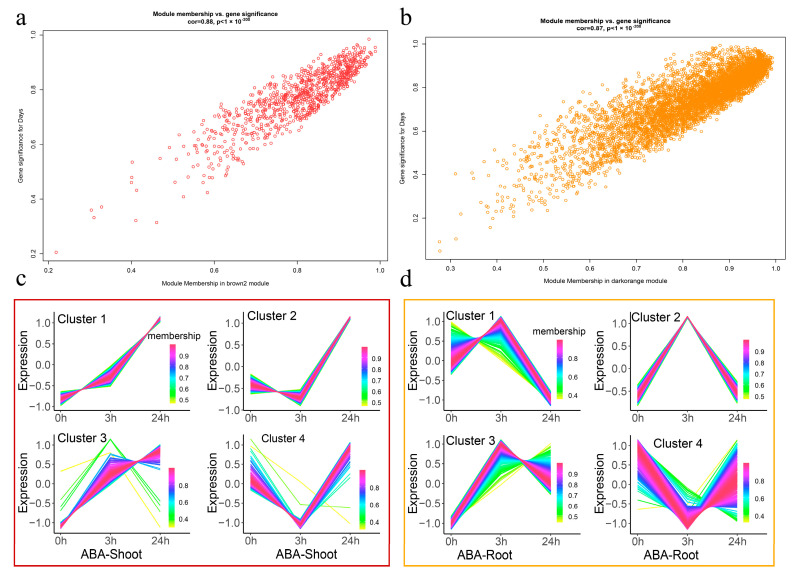
Gene significance (GS) and module membership (MM) correlation in the darkorange (**a**) and brown2 (**b**) modules. Expression profile analysis of DEGs in brown2 (**c**) and darkorange (**d**) modules at shoots or roots.

Among the 21 modules, the darkorange and brown2 module had relative high correlation coefficients (0.91 and 0.96) and presented significant associations (*p* < 0.001) with 3 h _root and 24 h_shoot, respectively (Figure 3), which indicated that the two modules had great relevance in terms of ABA treatment. Meanwhile, a significant correlation was observed between module membership (MM) and gene significance (R = 0.87, *p* < 1 × 10^−200^; R = 0.88, *p* < 1 × 10^−200^) in the darkorange and brown2 modules, respectively (Figure 4a,b). The above results indicated that the darkorange and brown2 modules were key modules in the gene network of ABA responsive genes.

To view the detailed expression patterns of genes in darkorange and brown2 modules, a heatmap of the gene expression level was conducted (Supplementary Figure S3). Overall, most of the genes in the darkorange module were upregulated in the root after 3 h ABA treatment, and most of the genes in the brown2 module were upregulated in the shoot after 24 h ABA treatment (Supplementary Figure S3). To further examine the dynamic expression changes of DEGs from 0 h to 24 h, a cluster analysis was performed for the genes in darkorange and brown2 modules. In the brown2 module, four clusters were found, two of which presented a down-regulated trend, and then an up-regulated trend compared with 0 h. The other two clusters showed a continuous up-regulated trend in the shoot (Figure 4c). In the darkorange module, four clusters were also found, of which three exhibited an initial up-regulated followed by a down-regulated trend, and one showed an initial down-regulated followed by an up-regulated trend in the root (Figure 4d).

### 2.4. Functional Enrichment Analysis of Two Modules

**Figure 5:**
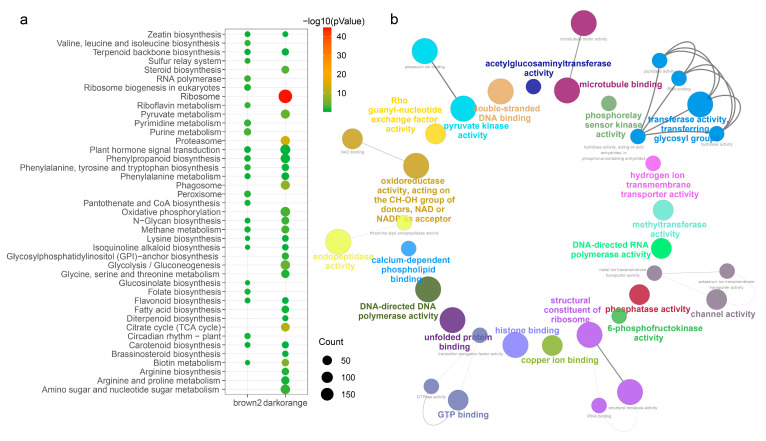
KEGG (**a**) and GO (**b**) enrichment analysis of genes in darkorange and brown2 modules.

GO and KEGG enrichment analyses were made for the genes in the darkorange and brown2 modules, with the significant terms of each category presented in Figure 5. KEGG enrichment analysis revealed that genes within the darkorange module were predominantly enriched in pathways related to protein synthesis and metabolism and secondary metabolites including proteasome, ribosome, biotin metabolism and steroid biosynthesis. And genes within the brown2 module were predominantly enriched in energy substance metabolism-related pathways including purine metabolism, pyrimidine metabolism, and phenylalanine, tyrosine and tryptophan biosynthesis. Furthermore, the two modules shared secondary metabolites pathways, like plant hormone signal transduction, phenylpropanoid biosynthesis, flavonoid biosynthesis and carotenoid biosynthesis (Figure 5a). The KEGG pathway enrichment results were in accordance with the GO molecular function (MF) enrichment analysis. GO-enriched results suggested that genes within those two modules were enriched in several pathways, primarily related to transferase activity, polymerase/phosphatase activity and GTP/DNA binding (Figure 5b).

**Figure 6:**
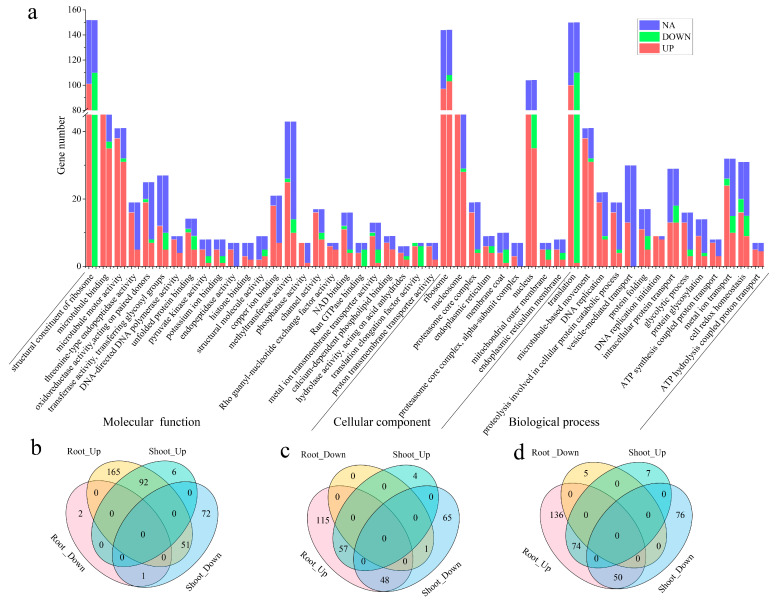
GO enrichment analysis of DEGs in the darkorange module under ABA treatment. (**a**) Genes within the darkorange module were significantly enriched (*p* < 0.05) in GO terms. The left and right of each GO term are the respective numbers of genes found in the roots and shoots. (**b**–**d**) Venn diagram of DEGs significantly enriched (*p* < 0.05) in GO terms from the shoots and roots of *M. ablus* under ABA treatment.

Genes in the darkorange module were significantly (*p* < 0.05) enriched into 48 GO terms, which are detailed in Supplementary Table S2. The stress and secondary metabolites-related main categories for molecular function were transferase activity, transferring glycosyl groups (GO:0016757) and methyltransferase activity (GO:0008168). The cellular components were endoplasmic reticulum (GO:0005783), mitochondrial outer membrane (GO:0005741) and endoplasmic reticulum membrane (GO:0005789), and the biological process was cell redox homeostasis (GO:0045454) (Figure 6a). To further investigate the similarities and differences in gene function between shoots and roots, the number of DEGs and non-differentially expressed genes (nDEGs) in each GO term were counted. The results showed that there were 389 DEGs in the molecular function category, 348 DEGs in the cellular component category, 290 DEGs were found in the biological process category and the up-regulated genes number in the root was more than that in the shoot (Figure 6b–d). We observed a few special GO terms, including the structural constituent of ribosome (GO:0003735), endopeptidase activity (GO:0004175), Ran GTPase binding (GO:0008536), vesicle-mediated transport (GO:0016192), and the genes expression pattern presented that, except for nDEGs, all DEGs were up-regulated in the root and down-regulated in the shoot (Figure 6). In addition, genes were annotated in GO terms, DNA-directed DNA polymerase activity (GO:0003887), threonine-type endopeptidase activity (GO:0004298), phosphatase activity (GO:0016791), proton transmembrane transporter activity (GO:0015078), calcium-dependent phospholipid binding (GO:0005544), ATP hydrolysis coupled proton transport (GO:0015991), ATP synthesis coupled proton transport (GO:0015986), etc., up-regulated except nDEGs in both the shoot and root. Genes in brown2 were enriched in GO terms, including biosynthetic process (GO:0009058), phospholipid biosynthetic process (GO:0008654), their response to hormones (GO:0009725), GTP binding (GO:0005525) (Supplementary Figure S4 and Supplementary Table S3). These results indicated that *M. ablus* responded to ABA treatment by regulating the genes associated with the three GO categories.

**Figure 7:**
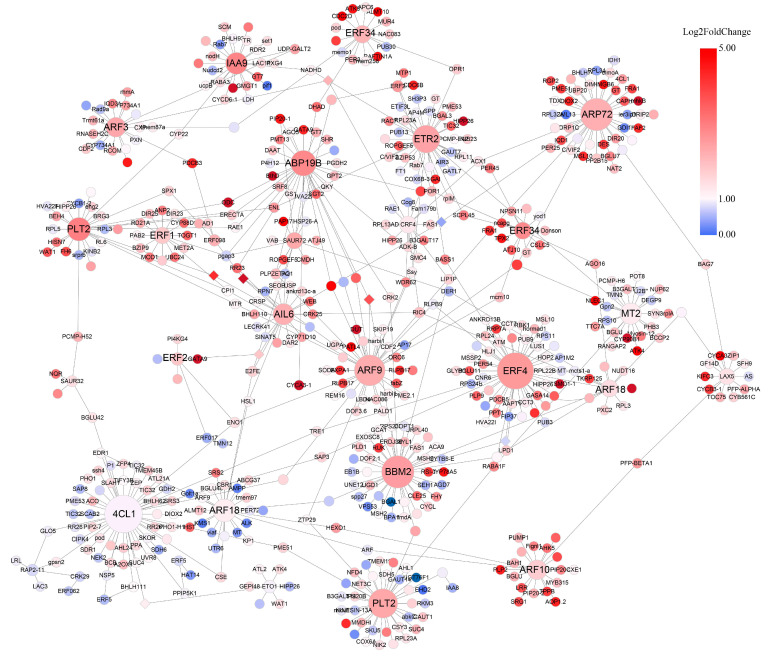
Co-expression network in *M. albus* under ABA treatment. Nodes and edges are represented by cycles and lines coated with color, respectively. The bigger the cycles, the higher the number of edges. Colors represent the base 2 logarithmic values of foldchange (3 h divide 0 h), color scale represented the values from 0 (blue) to 5 (red).

To narrow down the genes related to ABA treatment in the darkorange module, we annotated members and selected hormone-related genes. As a result of weighted co-expression network analysis, a network that included 616 genes was constructed. Among them, 567 genes had up-regulated expression and two genes had down-regulated expression (|foldchange| > 0.5, Figure 7 and  Supplementary Table S4). We found that most of the genes in the co-expression network were annotated as hormone and secondary metabolite related genes, and that among them, the hub nodes were three AP2-like ethylene-responsive transcription factors (AIL6, BBM2, PLT2), six ethylene-responsive transcription factors (ERFs), five auxin response factors (ARFs), 4-Coumarate: CoA ligase 1 (4CL1), auxin-binding protein 19B (ABP19B), auxin-responsive protein 72 (ARP72), ethylene receptor 2 (ETR2) and auxin-responsive protein (IAA) (Figure 7 and Supplementary Table S4). This result suggested that these hub genes played a core role in the regulatory networks of *M. albus* under ABA treatment.

**Figure 8:**
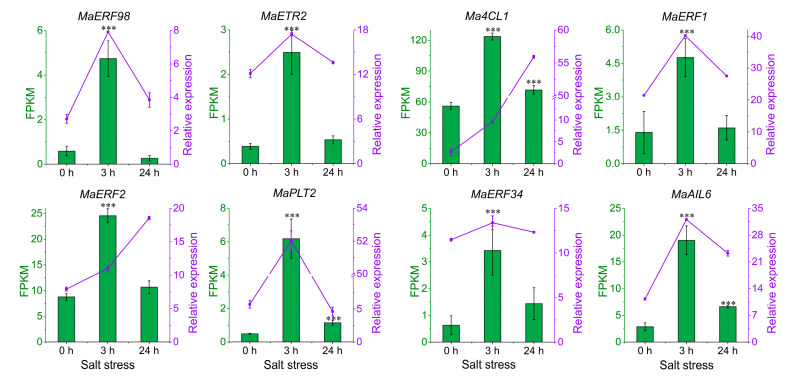
Expression profiles of ABA treatment responsive genes. The six-weeks-old *M. albus* plants were applied to 100 μM ABA. The qRT-PCR expression profiles (purple line) were consistent with the RNA-seq data (green bar) under ABA treatment in the root. Data are the mean ± SE (n = 3). ***, *p* < 0.001.

Hub genes within the network were selected for qPCR validation to verify their expression patterns. The overall expression trend of these genes was first increased and then decreased. There were six genes that presented a higher expression level at 3 h, and five genes presented a higher expression level at 24 h compared with 0 h after ABA treatment. The expression levels of these genes were validated by qRT-PCR, which provided results consistent with the RNAseq data for the *ERF98*, *ETR2*, *ERF1*, *PLT2*, *ERF34*, *AIL6* (Figure 8). Meanwhile, the expression trends of *4CL1*, *ERF2* showed a continuous increase after ABA treatment (Figure 8).

### 2.5. Gene Structures Analysis of Ma4CL Gene Family

**Figure 9:**
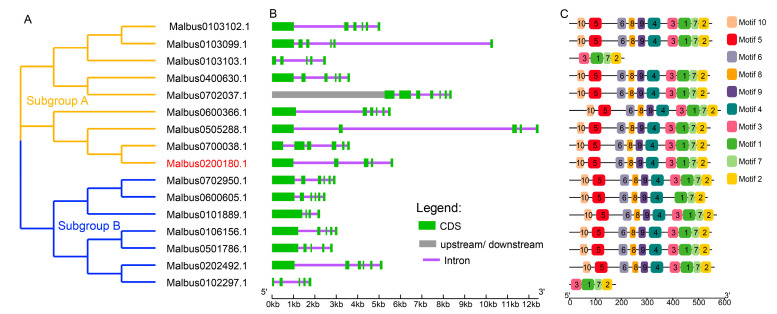
Phylogenetic relationships (**A**), gene structure (**B**) and identification of conserved motifs (**C**) of Ma4CL gene family. (**A**) The phylogenetic tree was constructed using a Ma4CL proteins sequence by MEGA 7 software based on the neighbor-joining method (1000 bootstrap replications). Subgroups are shown in different colors. (**B**) The exon–intron structure of Ma4CL members. Green boxes are exons, purple lines indicate introns, gray boxes represent the UTR regions. (**C**) The motif composition of Ma4CL proteins.

Previous research reported that the 4CL family in *M. albus* contains 17 members [36]. We identified 16 members among them in the present study, and through an analysis of their phylogenetic relationships, conserved motifs and the gene structure of them were discovered (Figure 9). The *M. albus* 4CL members were divided into two clusters (which were named subgroup A and subgroup B) based on a bootstrap test analysis (Figure 9A), and the result was similar with *Solanum tuberosum* [25]. Members in subgroup A contained five to seven exons, and in subgroup B they contained four to six exons (Figure 9B). A total of 10 conserved motifs were identified within the Ma4CL gene family (Figure 9C). All Ma4CLs contained motifs 3, 1, 7 and 2; among them, motifs 1, 7 and 2 belonged to AMP-binding regions. Furthermore, all proteins (except to Malbus0103103.1 and Malbus0102297.1) contained motif 6, which harbors the conserved amino acid region SGTT-PKGV and constitutes a pivotal component within the AMP-binding enzyme domain (Supplementary Figure S5). Subcellular localization prediction of Ma4CL indicated that proteins were distributed in the cytoplasm, plasma membrane and chloroplast (Supplementary Table S5).

### 2.6. Ma4CL1 Enhanced Tolerance of Transgenic Yeast to ABA and Abiotic Stresses

**Figure 10:**
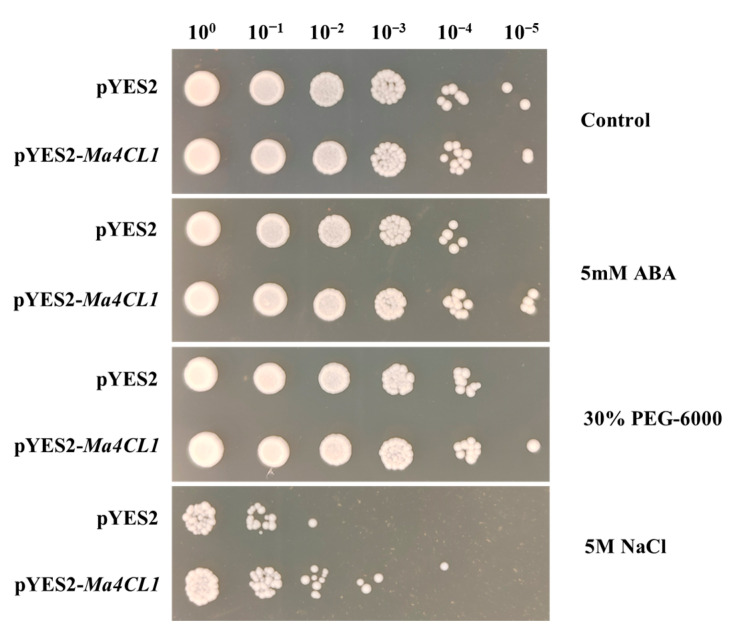
Analysis of ABA and abiotic stress resistance of *Ma4CL1* in a yeast expression system. Yeast harboring an empty pYES2 vector served as a control.

To verify the functions of *Ma4CL1*, we analyzed its effects on yeast growth and resistance to stress (including ABA, drought, and salt) in yeast cells containing the pYES2-*Ma4CL1* construct (Figure 10). The results indicated that both *Ma4CL1* transgenic yeast cells and yeast cells transformed with empty vectors (EV) exhibited similar growth under control conditions, with no discernible difference observed. The yeast cells transformed with *Ma4CL1* still grew under 10^5^-fold dilution, which indicated that transformed yeasts were not impacted by 30% PEG-6000 treatment. Meanwhile, the transformed *Ma4CL1* gene yeast cells presented prominent resistance under 5 mM ABA and 5 M NaCl treatments, especially under 10^5^-fold dilution for 5 mM ABA treatment and 10^2^-fold dilution for 5 M NaCl treatment (Figure 10). Therefore, we inferred that *Ma4C1L* plays a vital role in *M. albus* responses to ABA and salt stress.

### 2.7. Overexpression of Ma4CL1 Enhanced the Salt Tolerance of M. albus

**Figure 11:**
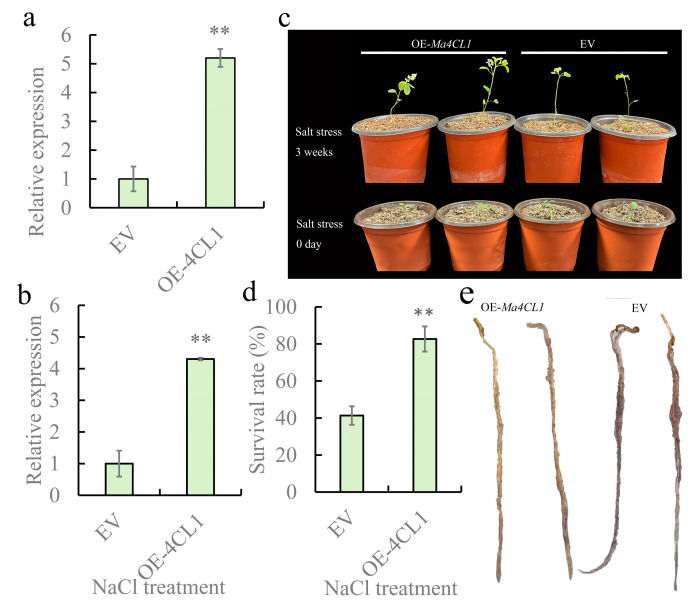
*Ma4CL1* positively regulated salt tolerance of *M. albus*. (**a**) Expression levels of *Ma4CL1* in EV and OE-*Ma4CL1* hairy roots. (**b**) Expression levels of *Ma4CL1* in EV and OE-*Ma4CL1* hairy roots under NaCl treatment. (**c**) The phenotypes of individual seedlings of OE-*Ma4CL1* and EV transgenic hairy roots which were observed after growing three weeks under normal conditions and NaCl treatment. (**d**) The survival rate of EV and OE-*Ma4CL1* after NaCl treatment. (**e**) Histochemical staining with NBT was performed on OE-*Ma4CL1* and EV hairy roots after three weeks of NaCl treatment. The error bars represented the standard deviation (SD) values calculated from the mean of three replicates. **, *p* < 0.01.

To further explore the regulatory role of *Ma4CL1* in stress tolerance, we conducted overexpression (OE) experiments through *Agrobacterium rhizogenes*-mediated hairy roots transformation. The positive transgenic hairy roots were verified by PCR (Supplementary Figure S6). The relative expression of *Ma4CL1* was confirmed by qPCR, and the results indicated that the relative expression of *Ma4CL1* had a significant (*p* < 0.01) increase in the positive OE-*Ma4CL1* transgenic hairy roots (Figure 11a). In addition, we found that the expression of *Ma4CL1* was induced by salt treatment (Figure 11b), and that overexpressed *Ma4CL1* in *M. albus* could improve the salt tolerance of transgenic plants (Figure 11c). The survival rate of the OE-*Ma4CL1* transgenic was 50% higher than that of the EV lines (Figure 11d). Nitroblue tetrazolium (NBT) staining results revealed that hairy roots with transformed EV exhibited more severe damage compared to OE-*Ma4CL1* hairy roots (Figure 11e).

## 3. Materials and Methods

### 3.1. Plant Materials, ABA Treatments and Physiological Indicators Measurement

Seeds of *M. albus* (JiMa46) were sourced from Lanzhou University. They were sown in flower pots filled with vermiculite. The upper and lower caliber was 9 cm and 7 cm, each measuring 11 cm in length, and they were watered by a Hoagland nutrient solution every week. The plants were placed in a growth chamber under 28/23 °C (day/night) temperatures, 50% relative humidity, 150 μmol quanta m^−2^ s^−1^ irradiance, and a 16/8 h (light/dark) photoperiod [7]. When the plants were six weeks old, a solution containing 100 μM of abscisic acid (ABA) was sprayed onto their shoots. A tiny sprayer was utilized to apply the ABA solution to the shoots until the entire plant was thoroughly wetted. The shoot and root were collected at 0 h, 3 h, 12 h, 24 h, and 48 h following the treatment, respectively, and then they were frozen in liquid nitrogen and stored at −80 °C. Physiological indicators of these samples were measured, and transcriptome sequencing was conducted for the samples at 0 h, 3 h and 24 h. Each sample was repeated three times. The proline, H_2_O_2_, and soluble protein content were determined using a kit (Solarbio, Beijing, China) based on the corresponding instructions.

### 3.2. RNA Extraction, Library Construction and Sequencing

The total RNA of each sample was extracted using TRIzol Reagent (Invitrogen, Waltham, MA, USA), following the instructions. The concentration of the RNA was measured by Nanodrop ND-1000, and RNA purity was performed using agarose gel electrophoresis (1.2%).

The RNAs were reverse transcribed into cDNA for constructing cDNA libraries, which were sequenced on a paired-end HiSeq2500 sequencing platform, with a read length of 125 base pairs. HISAT2 was used to aligned clean reads to the *M. albus* reference genome [37]. The FPKM (fragments per kilobase of exon model per million mapped reads) value for each gene was calculated based on the gene length and the reads count mapped to that gene [38]. StringTie 1.3.1 was used to quantify the expression values of each gene based on the FPKM [39]. Differentially expressed genes (DEGs) across various samples were identified using the DESeq2 1.46 software [40].

### 3.3. Functional Annotation and Enrichment Analysis

The Kyoto Encyclopedia of Genes and Genomes (KEGG) pathway enrichment analysis was performed using the KEGG Orthology-Based Annotation System server to assess the statistical significance of the DEGs enrichment [41].

The GOstat 2.72 tool [42] was utilized to perform gene ontology (GO) annotations for modules across the categories of biological process (BP), cellular component (CC) and molecular function (MF). A ClueGO 2.5.10 plugin was applied in Cytoscape 3.9.1 for visualization of GO enrichment.

### 3.4. Gene Co-Expression Network Analysis

According to the result from each sample regarding the gene expression quantity, and from sample clustering to detect potential outliers, we removed one root sample 24 h after ABA treatment (Supplementary Figure S7). Therefore, a total of 17 RNA-seq data under ABA treatment were used to co-expression analysis. The R 4.4.0 package was utilized to perform weighted correlation network analysis (WGCNA) for constructing gene co-expression networks [43]. An applicable soft-thresholding power, determined based on a scale-free topology criterion, was employed to convert the correlation matrix into a signed weighted adjacency matrix by computing the Pearson correlation coefficients between genes. The adjacency matrix served as the basis for computing the topological overlap matrix (TOM). Subsequently, all genes were hierarchically clustered based on their similarity using the dynamic tree cut method, resulting in the formation of modules, and the minimum module size was 40 genes. To address the correlation within pairs in data from a paired design, we employed a linear mixed-effects model to test for the association between a module and ABA treatment. Modules that were significantly (*p* < 0.01) related to ABA treatment were selected for further analysis in this study. Cytoscape 3.9.1 (https://cytoscape.org/, accessed on 23 November 2022) was used for network visualization. R 4.4.0 package “TCseq” was used to conduct a gene expression pattern analysis.

### 3.5. Quantitative Real-Time PCR Analysis (qPCR)

The total RNA of *M. albus* shoots was extracted utilizing the TRIzol Reagent (Invitrogen, USA). The 1st Strand cDNA Synthesis Reagent Kit, provided by Yeasen (Shanghai, China), was used to reverse transcribe RNA into cDNA. Eight DEGs were selected for qPCR verification. The primers specific to these 12 genes were designed (Supplementary Table S6). The expression level was normalized relative to the housekeeping gene *Tubulin*. qPCR was conducted, on the CFX96 Real-Time PCR Detection System (Bio-Rad, Los Angeles, CA, USA) using the Hieff qPCR SYBR Green Master Mix (No Rox) from Yeasen, following the instructions. The relative expression levels were calculated using the 2^−ΔΔCT^ method, and three biological repetitions were performed for each reaction.

### 3.6. Heterologous Expression of Ma4CL in Yeast

The coding sequence of *Ma4CL* were amplified using the cDNA of JiMa46 as the template. *Ma4CL1* was inserted into the pYES2 expression vector utilizing the ClonExpress^®^ MultiS One Step Cloning Kit (provided by Vazyme Biotech Co., Ltd., Nanjing, China), according to the provided protocol. The primer specific to *Ma4CL1* is detailed in Supplementary Table S1. The empty pYES2 plasmid and the recombinant pYES2–*Ma4CL1* were transformed into the *Saccharomyces cerevisiae* strain INVSc1, respectively, after sequence and PCR confirmation (Supplementary Figure S8). The yeast cultures were individually grown in synthetic complete (SC)–Ura liquid medium supplemented with 2% (*m*/*v*) glucose until A600 up to 0.4. Then, the yeasts were collected and incubated with SC-Ura containing 2% galactose until the A600 = 1. Subsequently, the yeasts were collected for stress analysis. An equal number of yeast cells were resuspended in solutions containing 5 mM ABA, 5 M NaCl, and 30% PEG-6000, respectively, and incubated at 30 °C for 36 h. Simultaneously, an equal quantity of yeast cells was resuspended in sterile water to be used as a control group. The treated yeast cells were diluted to 10^0^, 10^−1^, 10^−2^, 10^−3^, 10^−4^, 10^−5^, respectively, and then 2.5 μL of each was dispensed and spotted onto SC–Ura agar plates. These plates were incubated at 30 °C for three days. Photographs were then taken to assess colony formation and growth.

### 3.7. Ma4CL1 Overexpression Vector Construction and Transformation into M. albus

To produce the *Ma4CL* overexpression (OE) constructs, the full-length cDNA of *Ma4CL1* was cloned into pBI121 binary vectors (Supplementary Table S6), using the ClonExpress^®^ MultiS One Step Cloning Kit. The constructs were introduced into *Agrobacterium rhizogenes* K599 strain via electroporation. Transgenic hairy roots of *M. albus* were obtained by following the previously established protocol [44]. Transgenic lines transfected with an empty vector (EV) served as the control group. The transgenic hairy roots were screened using PCR to identify the positive ones (Supplementary Figure S6). After one week, the positive plants were transferred to flowerpots filled with vermiculite for continued cultivation for three weeks.

### 3.8. Salt Stress and Histochemical Staining Measurement

Two-month-old *M. albus* transgenic plants were subjected to treatment with 200 mM NaCl for 3 weeks. The Hoagland nutrient solution containing 200 mM NaCl was poured over the vermiculite in the flowerpots until the solution drained out from the bottom, with this process being conducted once every 5 days. The treatment with Hoagland nutrient solution was set as the control. At least 25 OE and EV transgenic plants were assessed for each treatment, respectively, with all treatments being replicated three times. After the treatments were completed, the survival rates of the plants were recorded. The transgenic hairy roots were stored in liquid nitrogen for qPCR experiments, and fresh transgenic hairy roots were immediately collected for histochemical staining. The transcript level of *Ma4CL1* in the OE and EV transgenic hairy roots was detected by qPCR. Histochemical staining for O_2_^−^ was performed using the BCIP/NBT Chromogen Kit sourced from Solarbio (Beijing, China).

## 4. Discussion

In natural environments, plant growth and development are jointly influenced by both environmental factors and endogenous hormones. Therefore, in addition to cultivating excellent varieties with strong stress tolerance, researchers are exploring appropriate exogenous substances to treat seedlings, aiming to enhance their environmental adaptability. ABA plays a vital role in plants’ responses to adversity, including drought, soil salinity and cold temperatures [8,9,29], and applying ABA can enhance the abiotic stress resistance of plants [34,45]. However, the regulatory mechanism of ABA in *M. albus* has not yet been elucidated. Here, we comprehensively analyzed *M. albus* shoots and roots after ABA treatment. We found different response patterns of genes in shoots and roots, constructed an ABA-related network and analyzed the function of *Ma4CL* gene in vitro and in vivo. The results indicated that the data acquired by RNA-seq were reliable and identified 13,392 DEGs in shoots and 15,471 DEGs in roots. Our DEG analysis revealed that genes exhibit distinct responses in shoots and roots after ABA treatment, and that gene expression patterns can vary based on the duration of treatment.

The integrated RNA-seq and WGCNA analysis has become a crucial and economically effective approach for uncovering key genes and their interactions that are potentially functionally associated with specific traits [46]. By performing the correlation analysis of modules using time points and eigengenes, two modules significantly correlated with ABA treatment were chosen for further analysis in this study. We found that the expression of genes within the two modules responded to ABA treatment. Genes in the darkorange module were predominantly enriched in pathways associated with protein synthesis and metabolism and steroid biosynthesis, and genes in brown2 module were predominantly enriched in energy substance metabolism. Proteins have a direct effect in plants’ response to adversity, and the plant’s acclimation to stress is accompanied by significant alterations in proteome composition [47,48]. Plant sterols serve as crucial components of the lipid rafts and cell membrane which are regarded as a requirement for plants in response to abiotic stress [49]. Drought stress resulted in the increased content of sterols in rice, with the drought-resistant variety exhibiting a higher sterols content [50,51]. This result indicated that stress promotes steroid biosynthesis. Research has demonstrated that crosstalk between NAD(P)+ and ABA plays a role in regulating plant responses to abiotic stress [52,53]. An increase in NAD(P)+ can facilitate ABA biosynthesis and signal transduction, thereby boosting the plant’s responsiveness to ABA and stress; conversely, ABA signaling can inhibit further increases in NAD(P)+ levels [52,53]. Phosphoribosylation is a crucial step in the biosynthetic pathway of purine and pyrimidine nucleotides, as well as the cofactor NAD(P)+, serving to regulate the production of these metabolites [54]. Previous research discovered that salt-resistant plants exhibit faster purine production rates and slower degradation rates compared to salt-sensitive plants, which allow them to sustain significant levels of nucleic acids and nucleotides in response to salt stress [55]. By manipulating ABA levels and the associated signaling pathways, it is possible to develop plant varieties with enhanced productivity in adverse environments [56]. Therefore, the results in the present study indicate that the exogenous application of ABA activated the stress resistance system of *M. albus*, which benefited from an improved stress resistance.

Previous research has demonstrated that exogenous application of ABA enhances stress tolerance, as does the overexpression of genes, leading to an increase in endogenous ABA content in plants [34]. Exogenous application of ABA on *M. albus* resulted in a significant number of alterations in gene expression. Members in darkorange and brown2 modules may play positive roles in ABA response, given the up-regulated expression of them after ABA treatment, especially, hub genes *MaERF1*, *MaERF2*, *MaERF34*, *MaERF098*, *MaETR2* and *Ma4CL1* were upregulated after ABA treatment. It had been reported that *GmERF3* [57], *CsERF* [58], *JERF1* [59] and *LchERF* [60] were induced by ABA. Notably, *JERF1* interacted with cis-acting elements could activate both stress-responsive genes and ABA biosynthesis related to genes, ultimately leading to an enhanced salinity tolerance in tobacco [59]. It has been found that *Hc4CL* expression was induced by ABA treatment [26]. There was a similar result in the present study, in which the *Ma4CL* expression level was significantly upregulated after 3 h of ABA treatment in shoot. The activities of 4CL were associated with plant hormones and were highly dependent on the ratio of ABA/GA3 [61]. Overexpressed *4CL* can enhance the activity of CAT and POD and reduce levels of malondialdehyde, thereby improving salt and osmotic tolerance of transgenic tobacco [62]. In this study, *Ma4CL1* transgenic plants showed a higher salt tolerance, and histochemical staining of transgenic root hair showed lighter colors. The lower O_2_^−^ content observed in the transformants compared to the wild type indicated that the enhanced stress tolerance of the transgenic plants may be partially due to the increased activity of certain antioxidant enzymes.

## 5. Conclusions

In summary, the present study offers a comprehensive analysis of the transcriptome of *M. albus* in response to exogenous ABA application, highlighting the significant transcriptional variations that occur. ABA serves as a crucial hormone in plants’ response to stress, and our results revealed that the stress tolerance related pathway may be activated after exogenous application of ABA in *M. albus*. The extensive divergence observed in the transcriptome, encompassing differential gene expression patterns, KEGG metabolic pathways and GO functional analysis, indicated that the exogenous application of ABA may have a positive effect on inducing plant tolerance to environmental stress. We identified a co-expression network within the ABA-responsive pathways that could play an important role in improving abiotic stress tolerance in *M. albus*. Functional analysis of one hub gene, *Ma4CL1*, was performed in vitro and in vivo, and the results suggested that *Ma4CL1* plays a vital role in salt tolerance of *M. albus*. The data obtained from this study have highlighted numerous candidate genes associated with ABA signaling, offering potential target genes for molecular breeding to improve stress tolerance in *M. albus*.

## Data Availability

The raw data of RNA-seq are available from the National Center for Biotechnology Information Short Read Archive under bioproject PRJNA1120345.

## Supplementary Materials

The following supporting information can be downloaded at: https://www.mdpi.com/article/10.3390/ijms252413261/s1.
