# Supplementary material for: Comparative Transcriptome Analysis Revealing the Potential Salt Tolerance Mechanism of Exogenous Abscisic Acid Application in Melilotus albus
Source: Int J Mol Sci. 2024 Dec 10;25(24):13261. doi: 10.3390/ijms252413261 (PMC11676779; doi:10.3390/ijms252413261)
Supplement: Supplementary file 1 [file ijms-25-13261-s001.zip › Supplementary Figures S1-S8 and Tables S1, S5 and S6.pdf]

Supplementary Materials Information

Supplementary Table S1. Assessment of assembly quality for *M. albus* libraries of different sample.

Sample	Clean Reads	Clean Bases	Q30 Rate (%)	Mapping Rate
S_0h1	133,245,520	19,986,828,000	0.93	97.57%
S_0h2	169,153,864	25,373,079,600	0.92	97.50%
S_0h3	139,408,572	20,911,285,800	0.93	97.32%
R_0h1	137,283,886	20,592,582,900	0.94	85.97%
R_0h2	140,929,298	21,139,394,700	0.93	88.60%
R_0h3	144,789,250	21,718,387,500	0.93	88.00%
S_1h1	40,012,024	5,955,342,942	0.94	97.92%
S_1h2	39,841,726	5,937,204,808	0.94	98.04%
S_1h3	53,349,914	7,947,600,489	0.95	98.09%
R_1h1	44,678,818	6,638,150,429	0.94	96.77%
R_1h2	51,305,178	7,642,844,140	0.94	92.94%
R_1h3	42,157,806	6,283,408,456	0.94	96.16%
S_24h1	42,328,194	6,301,533,056	0.95	97.88%
S_24h2	39,906,706	5,925,675,508	0.94	97.68%
S_24h3	41,504,724	6,191,388,724	0.94	97.87%
R_24h1	40,438,774	6,036,329,514	0.94	76.94%
R_24h2	42,858,918	6,386,748,426	0.94	95.81%
R_24h3	41,729,954	6,216,955,701	0.94	92.40%

Supplementary Table S2. GO enrichment information of genes in darkorange module.

Supplementary Table S3. GO enrichment information of genes in brown2 module.

Supplementary Table S4. Abbreviation and function annotation of genes in the co-expression network.

Supplementary Table S5. Subcellular localization of Ma4CL gene families.

Query Protein	Predicted Location(s)
Malbus0106156.1	Plasma membrane
Malbus0202492.1	Chloroplast
Malbus0702950.1	Plasma membrane
Malbus0700038.1	Cytoplasm
Malbus0505288.1	Cytoplasm
Malbus0400630.1	Chloroplast
Malbus0600366.1	Cytoplasm
Malbus0200180.1	Cytoplasm
Malbus0102297.1	Cytoplasm
Malbus0702037.1	Chloroplast
Malbus0101889.1	Cytoplasm
Malbus0103102.1	Chloroplast
Malbus0103099.1	Chloroplast
Malbus0103103.1	Chloroplast
Malbus0600605.1	Plasma membrane
Malbus0501786.1	Plasma membrane

Supplementary Table S6. List of primers used in the present study.

Gene	Primer
qRT-PCR-*ERF34*-F	ATCAAAGCCTCTCCAAATGC
qRT-PCR-*ERF34*-R	GCTGTAGGGTATGTTCCAAGC
qRT-PCR-*AIL6*-F	CTGCTACCACCAACTTCCCT
qRT-PCR-*AIL6*-R	GTTGATGATGCCTTGTAACACC
qRT-PCR-*4CL1*-F	ATTCTTGGACAGGGATACGG
qRT-PCR-*4CL1*-R	GGTAAAGAATTGCCAGTTTCAG
qRT-PCR-*ERF98*-F	TCCAATAGGCAGGGTCAGAG
qRT-PCR-*ERF98*-R	GCATTCCTTGAAGATGAACCAG
qRT-PCR-*ETR2*-F	GAAGATAATCCAGTCAATGAGAGG
qRT-PCR-*ETR2*-R	CTACCAACACTTGCAGACCT
qRT-PCR-*ERF1*-F	GAAGAAATGAAGCACATGACAAGG
qRT-PCR-*ERF1*-R	ATGTTGATGATGTCTCGTTACTCC
qRT-PCR-*PLT2*-F	TTAGCTGCACTAAAGTATTGGG
qRT-PCR-*PLT2*-R	TGTTGATGATGCCTTGTGAC
qRT-PCR-*ERF2*-F	TCTGACTGAGGAGGAAGCAG
qRT-PCR-*ERF2*-R	CTCTGATGAAGAAGAAGAAGGCTC
qRT-PCR-*Tubulin*_F	CCTTGGTGGTGGAACTGGT
qRT-PCR-*Tubulin*_R	GGAGATGGGAACACTGAGAAAG
pYES2-*Ma4CL1*-BamH I-F	CTTGGTACCGAGCTCGGATCCATGGCGTCCAAACAAGAAAC
pYES2-*Ma4CL1*-Xba I-R	TACATGATGCGGCCCTCTAGATTAATCAATTTGGAACACCAGCTG
pBI121-*Ma4CL1*-Xba I-F	GAGAACACGGGGGACTCTAGAATGGCGTCCAAACAAGAAAC
pBI121-*Ma4CL1*-BamH I-R	CCATGGTACCCCCGGGGATCCGAATTTGGAACACCAGCTGC

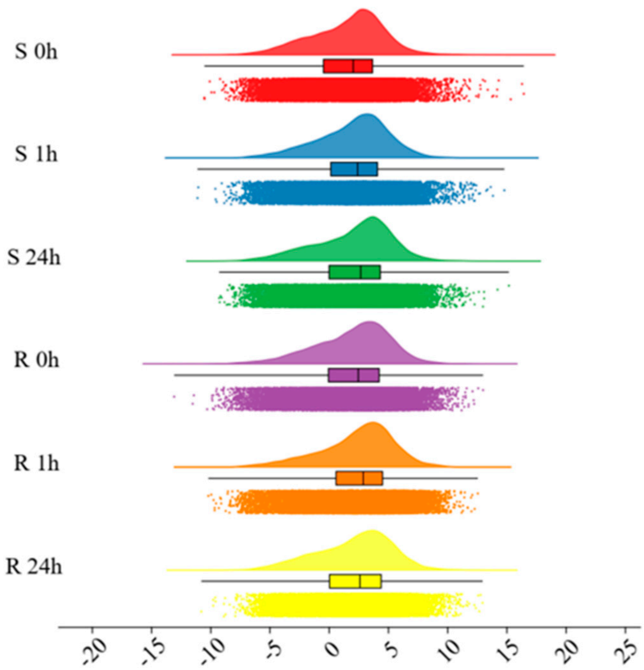

Supplementary Figure S1. Raincloud plots of different sample. All genes FPKM values of each sample were used to analyze.

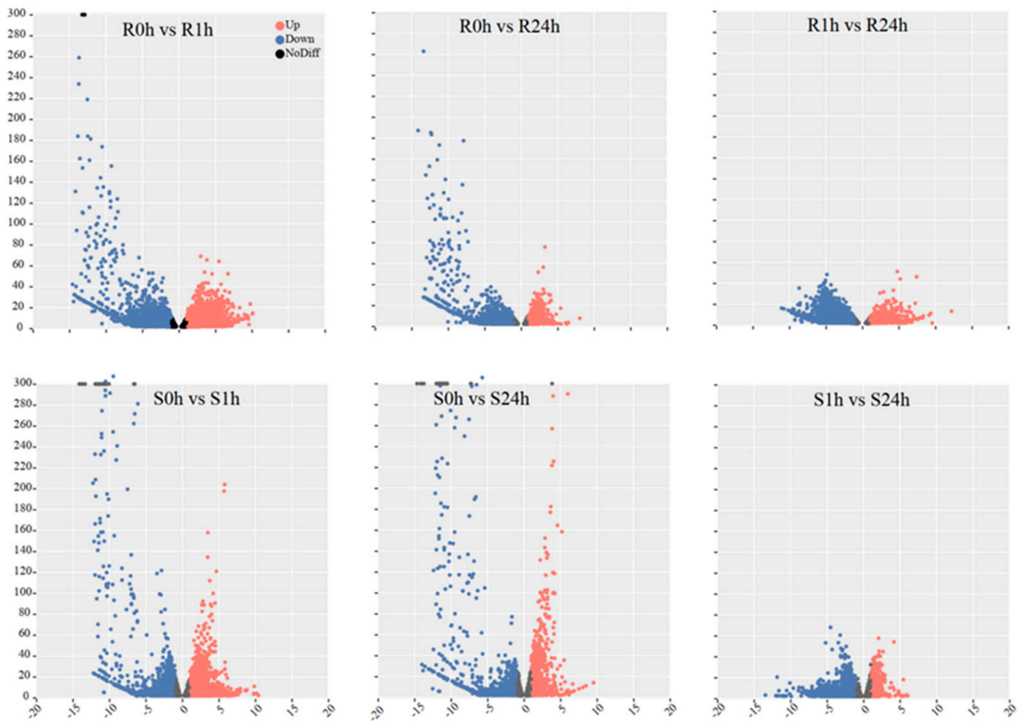

Supplementary Figure S2. Volcano maps showed overall differences between treatments.

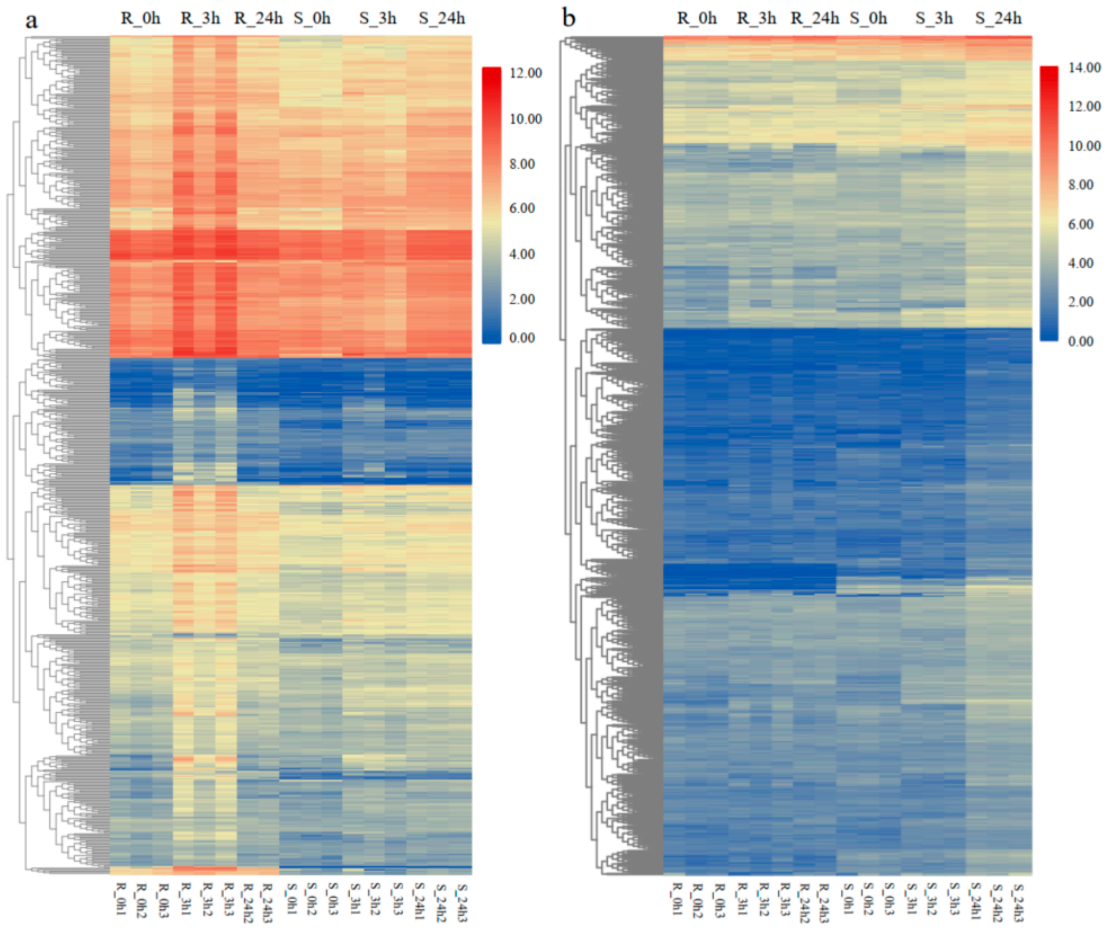

Supplementary Figure S3. Heatmap of orange (**a**) and brown2 (**b**) modules genes clustered by hierarchical clustering analysis.

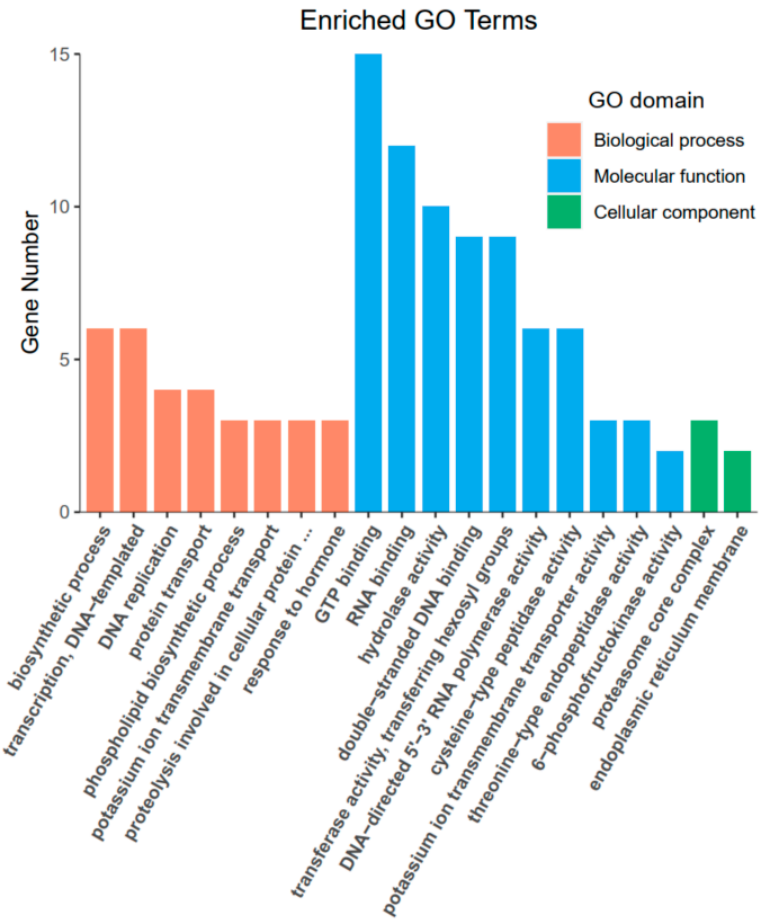

Supplementary Figure S4. Gene ontology (GO) enrichment analysis for all genes of brown2 module.

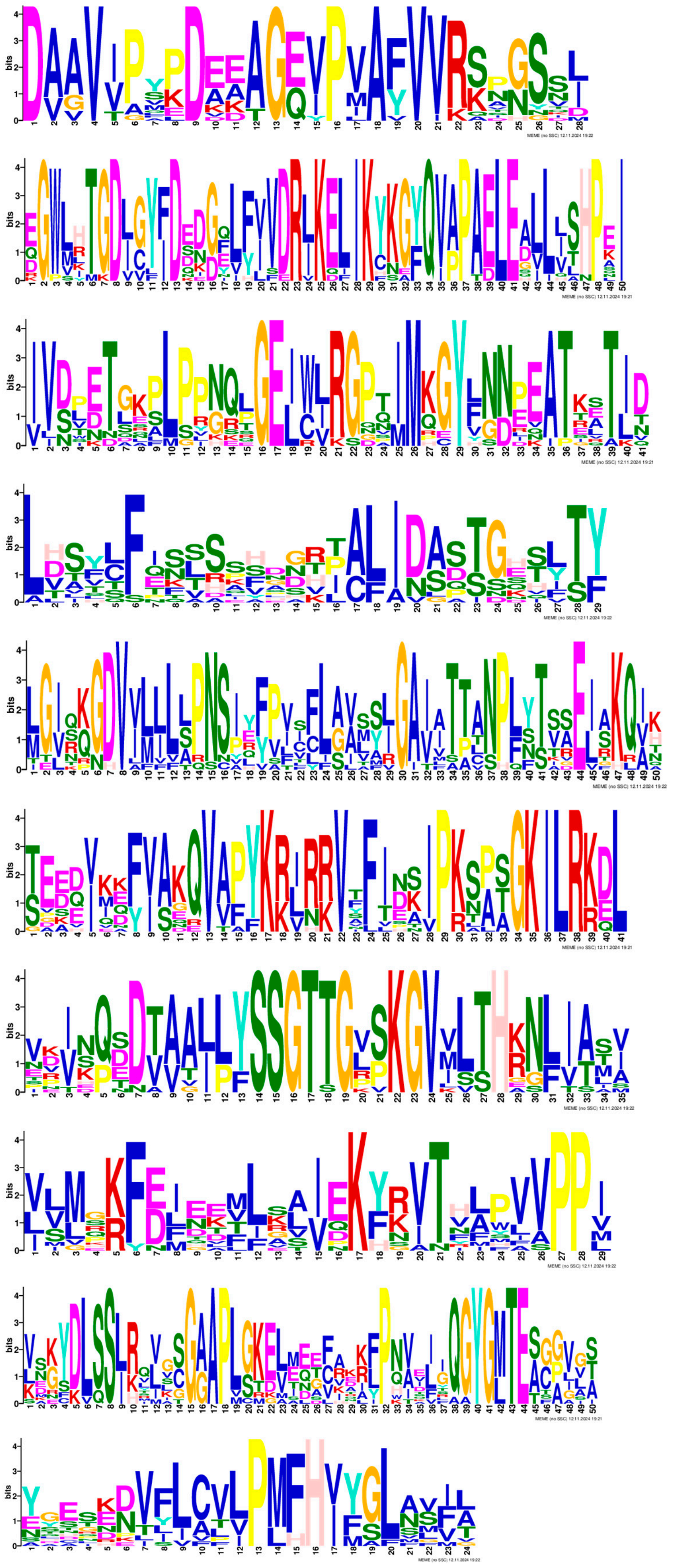

Supplementary Figure S5. The sequences of 15 motifs.

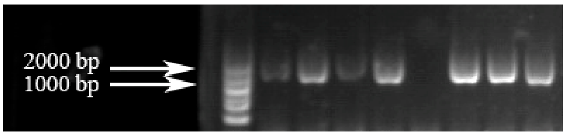

Supplementary Figure S6. The *Ma4CL1* positive transgenic lines verified by PCR.

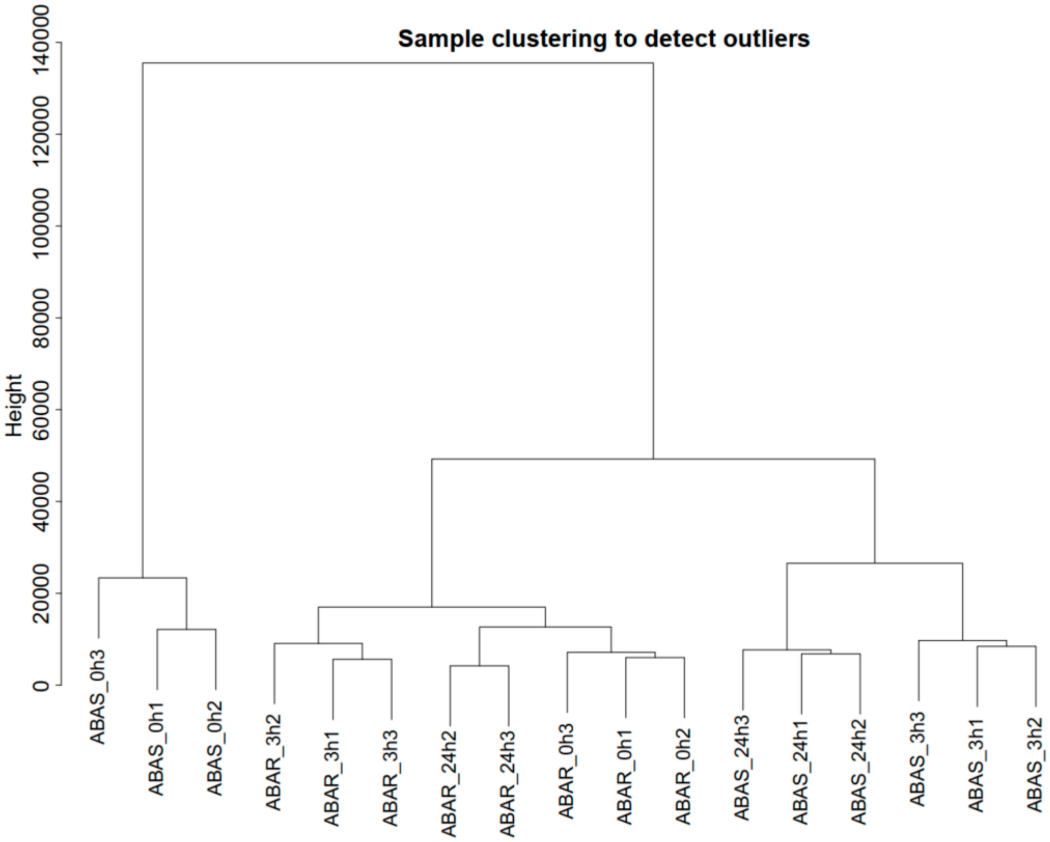

Supplementary Figure S7. Phylogenetic dendrogram of all 17 samples. ABAS: shoot after ABA treatment; ABAR: root after ABA treatment.

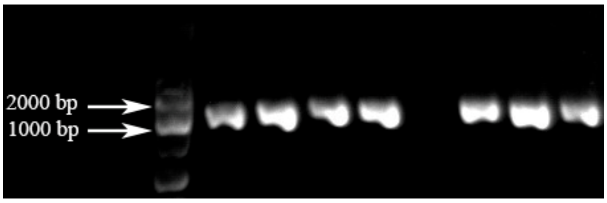

Supplementary Figure S8. The *Ma4CL1* positive yeast strains verified by PCR.
